# Observations on Some of the Operations of the Human Understanding

**Published:** 1863-07

**Authors:** John Alexander Davies


### Art. IV.—OBSERVATIONS ON SOME OF THE OPERATIONS OF THE HUMAN UNDERSTANDING.

By John Alexander Davies.

The human mind, mental and moral, is the only thing about man which he can call himself, and this both because nothing about his corporeal frame can be supposed to constitute his personality, and the mind alone is constant. It is sometimes said that knowledge should be pursued for its own sake; but, leaving this as a somewhat doubtful and not very clear point, it may be set down as something acknowledged by all, that every species of inquiry which appears likely to yield results conducive to utility, may lawfully be entered upon. The investigation of the various phenomena of the human mind may from experience be said to be within this restriction.

It appears reasonable to affirm that before the manner of the operations of the human mind is inquired into, the phenomena themselves should receive a close inspection, and this because the assumption of unproved causes with a view to the determination of effects is indirect, inasmuch as it may be necessary to suppose many causes, and also because in the majority of cases many effects are known, and the transition from supposed causes to others or observed effects would probably often be unjust. The phenomenon of perception is one which has not been explained in a way satisfactory to the whole of the philosophical community. Ordinary people have no difficulty upon the matter, and this of course because they never choose to give it their attention. No person, it may be presumed, ever supposed that the wall or tree before him existed in his mind when he looked upon it; but it is a general idea that we immediately behold the various objects surrounding us, although one, as has been observed, that the slightest philosophy dispels: the light constantly flying from off objects produces impressions of them upon the retina, which by means of nerve vibrations are carried into the brain; and perhaps here principally, if not entirely, the mind becomes affected. It is, and indeed could not but be granted, that we have an immediate knowledge of every mental affection or condition; but whether or not man has an immediate knowledge of more than this, is a point which has not been settled so as to meet with universal, or indeed general, approval. When I see a tree before me I know that my mind exists in a corresponding condition. I am immediately conscious of this; but, whether or not I am similarly conscious of the impressions upon my retinas, and of the existence of the tree, is, as it requires little attention to prove, a different question. I apprehend that an immediate consciousness of these things cannot be made out; or, which is the same thing, that the knowledge man has of them depends upon probability. When I see a tree, or a house, I conclude that these things are really existing, and that their impressions have necessarily been made upon my retinas; and this because I feel my mind affected similarly to what it has been the subject of, and conclude that its present condition is produced by similar causes; and when my eyes are directed to any object, I consider it very probable that the same effects are produced by the same causes, and thus conclude that it is real, and that its impression is produced upon my retinas. Now, inasmuch as it must be considered possible that the mind could exist in the condition it does when the eyes are gazing upon a tree, and thus when its impression is produced upon the retinas, apart from either of these conditions, it appears to me contradictory to say that man has an immediate knowledge of any object, its impression upon his retinas, or anything except the condition of his mind, or the proximate physical effect. I think it is generally supposed that man has a more immediate knowledge concerning—that is, is surer of the existence of these impressions, than of that of their objects; but, besides that the term immediate does not admit of degree, it should be considered that the connexion between the object and the mind is made by an unbroken chain of physical causes, and thus that the existence of any part of it is neither more or less certain than that of any other: if we have not the immediate perception of any object, neither have we that of its impression upon the retinas, although this part of the physical chain is nearer to that link which impresses the mind.

This consideration modifies or diminishes the popular error, which, as every person may at once perceive, arises not from thought but the entire want of reflection, when it is seen that the impression produced upon either retina is no more immediately perceived by us than is the object: because both these things form a chain of physical causes, it will at once appear that the popular idea, which takes no cognizance of these impressions, is no more unphilosophical than the so-called philosophical one, which holds that they are immediately discerned. If it is supposed that the want of the impression of external objects renders the ordinary idea the more ridiculous, it should be considered that these impressions end in working a number of vibrations throughout the brain, and that these vibrations might, as far as man can tell, be produced without them.

The distinction between sensation and perception is well known, and thus need not here be explained ; but it will be well to observe, if indeed it is not sufficiently clear, that during the perception of any object the mind exists in two states: first, as its sensation or impression if our feelings are divided into two sorts, which appears convenient; and, secondly, as the remembrance of having referred this to some inferred external object, or, if it be sensation proper, as the pressure of some heated surface upon the hand, or the prick of a pin, to some object in contact with oneself. This is the duality of consciousness, by which term I mean any single condition of the mind. To say that the mind is immediately conscious of anything but its own condition at the time, or, which is the same thing, that it immediately perceives material qualities, could not be pronounced an impossible idea were we sure that its various conditions could be produced in several ways; but, inasmuch as it cannot absolutely be proved that the supposed impressions upon the retina are conditions of the mind arising from the vibrations proceeding from such and more distantly from corresponding objects, we cannot say that we are immediately conscious of any physical effect. I do not see that to suppose an immediate perception of physical effects is to suppose the identity of matter and mind, and this because the perceiving body must be of the same nature as the phenomenon perceived, inasmuch as, as far as I can see, this assertion is one unattended by any proof, and yet brought forward on many occasions as something evident when required. The immediate perception of physical effects cannot be resisted on the ground that the mind knows such only as they alter its condition, because this affirmation cannot be proved: we do not know that the mind does not apprehend physical phenomena in an immediate manner, that is, apart from any alteration of its condition ; but inasmuch as we are unable to say that various physical effects are not ultimately at different times produced in different ways, I say in that we are unable absolutely to demonstrate this by the aid of human reason, we cannot, even on the hypothesis of the immediate perception of proximate physical effects, affirm that we have the immediate perception of anything beyond, of inferred external objects, than impressions upon the retina, or any other parts of the chain of physical causes, that is, in relation to their effect upon the mind, whether their influence is supposed to be immediate or conditional as altering its condition, and thus representatively perceived. It is a well-known scientific axiom that similar effects must be produced by similar proximate causes; but this, of course, cannot reasonably be set up against the doctrine, that the ultimate cause and intermediate causes of similar effects are frequently very different. Scores of contrivances could be adopted for the purpose of moving a carriage along a road at a given rate: here the proximate effects would be motion and friction, and the ultimate and intermediate causes would be various. It may be thought that the idea of the immediate perception of physical effects acknowledges effect to the exclusion of a corresponding cause, that if physical effects do not alter the condition of the mind, they cannot be supposed to affect it in any other way; but this idea cannot, I think, be made good by any sort of proof, nevertheless it is evidently one which should receive our closest attention. If it is granted that the mind immediately perceives proximate physical effects, it may be considered whether it at the same or any other time becomes acquainted with these representatively. I do not think that the immediate perception is, supposing it to exist, an active condition, although it must be supposed to differ from a change of mental condition, not as a phenomenon diverse in degree but in kind. It may be supposed that the doctrine of the immediate perception of the last chain of any physical effect shows the door for the absolute proof of the existence of external objects; but this I apprehend is going too far: for it cannot be shown impossible that these effects, supposing them to be immediately perceived, are spiritually caused, and thus do not proceed from physical objects. When we remember how great the probability that the various conditions of the mind, which are supposed to be produced by physical objects and phenomena, or the various physical effects which it may be supposed are immediately perceived, are owing to physical objects and phenomena, both of course by the same chain of physical causes, the supposed danger of slipping into idealism will disappear, and be esteemed as something which will never prove a quagmire to any but the most perverted mind. There are some persons who, however improbable any opinion, seem determined to believe it to be true until its falsity is demonstrated; so that as regards some things which are universally considered to be facts, they never profess belief. Atheists and many sceptics are men of this class; and it is to idealists, if it is true that such persons ever have existed, which I cannot consider credible, and alone to persons who do not consider the amount of probability in favour of any doctrine as contrasted with that against it, and found their belief upon that, but who appear blind to the comparatively unfavourable amount of probability in favour of the doctrine that pleases them, that the doctrine of representative perception, or the immediate perception of one link of the physical chain proceeding from any single object, can be considered dangerous. When speaking of atheists, I did not mean it to be inferred that the existence of God cannot absolutely be proved, for holding the doctrine of the eternity of matter being something only very improbable to be absurd, I maintain that it is capable of absolute proof; I only meant to say that because atheists fancy the existence of God incapable of absolute proof, they morbidly choose, that is, as far as profession goes, to pass over what they allow to be the probability, and, perhaps in some cases, the superior probability, in favour of this, and thus refuse to give their assent to it; what they think being, of course, another thing.

Except as the mind in any of its single, active, or passive states, that is, as existing in the condition of memory or any other active state, or as in some way passively perceiving inferred impressions and sensations, I cannot find that the term consciousness is suggestive of any meaning; so that to those who consider consciousness to be something different to these things, and thus, as it seems to me, necessarily a separate faculty, I can only say that I do not understand them, and am compelled, judging from what I find in myself, to believe their doctrine to be erroneous. Those who well consider it will be ready to allow how likely we are in all investigations connected with the mind, to be misled with words: many current terms have no fixed meaning, being used in various senses by different persons, and sometimes variously by the same individual; nevertheless, whenever a general term is used, the person making use of it has a general idea corresponding thereto, although of course in the generality of cases these ideas are very loosely formed. The term consciousness, though not so commonly used as is conscience, must still be considered a popular one, and according to its ordinary meaning signifies either memory or the knowledge of the mind at the present time, whereas the word conscience is generally applied to the moral part of man; but, although I believe that consciousness is always used in one of these senses when ordinarily employed, yet it seems to me that there is even a vague notion of its being something separate, contradictory as this appears. If we ask our friends to tell us what they mean by the term consciousness, we shall find that their answers may be resolved into one of the two conditions which have been given ; and yet if it is noticed how the word is brought out in ordinary conversation and society, one cannot help supposing that a separate mental faculty is intended. It may be that reflecting minds have stumbled and are now perplexed at this, and thus that the consideration of consciousness, as a separate faculty of the mind, is found in the records of philosophy.

A recent philosopher has observed, that the hypothetical realist, when asked how he proves the reality of the outer world, can only say that he infers its existence from the fact of its representation ; but I am unable to understand that those who hold the doctrine of representative perception are tied to this answer. A person of this persuasion can say that from the condition of his mind, when his eyes are open, he infers that the last link of a chain of physical causes creates it, and that these proceed from external objects. It is impossible upon this doctrine to do more than infer the representations and external objects, and consequently no absurdity can be turned against it by considering these things to be assumed as facts or truths which can be demonstrated, so that the remarks of the same philosopher—" But the fact of the representation of an external world supposes the existence of that world, therefore he is again at the point from which he started,"—though conclusive against it as thus put, are not applicable to the real doctrine. And this, I think, will at once be seen. Neither are they applicable to the supposition that the mind immediately perceives the last link of the chain of physical effects, and thus because as before the representations and existences of external objects are considered not as demonstrated but only as inferred facts. I know that revelation has been supposed to set aside idealism, but cannot say whether an apodictical or demonstrative in opposition to an inferible or only probable proof, using the former term in its ordinary sense, has been given. It appears to me that revelation affords a demonstrative refutation of idealism : a man with the Bible in his hand may read that God made the world, and he may go on to peruse the description of the same, and some of the remarkable events which characterized the ancient ages. Now, if he holds the doctrine of representative perception, he will, if he is convinced of the truth of that which men call the Bible, be bound to believe that the several conditions of his mind have occurred according to the will of God ; and thus that these conditions imply realities, inasmuch as we are unable to suppose that God is deceiving us. And the same thing applies to the other doctrine which has been noticed. If a person thinks that he immediately perceives the last link of any chain of physical effects, he will, if he believes the Bible, be found also to believe that this link is both caused by a real book, and demonstrates the existence of external objects and phenomena. I cannot find any reason against supposing that the mind exists in states embracing—of course simultaneously, for the term state implies this—the consciousness of a plurality of things. I may have the impression of St. Paul's, and at the same time perceive it, or become impressed with the fact of its existence. Why it has been supposed that man cannot be conscious of more than one thing at a time, apart from the doctrine of the soul's indivisibility, and the consideration of some other qualities which have been applied to it, I cannot tell, for I take everything in the way of experience to prove that the mind can be variously impressed simultaneously.

I confess that the doctrine of the various so-called impressions produced upon the mind being only itself in certain states, appears to me the only conclusion which our evidence enables us to make, and I cannot find any real objection to it. Were I disposed to make use of the term common sense, I should apply it to this doctrine; but as it appears that any distinction between reason and common sense is misplaced, and, moreover, widens the gap between the affairs of common life and the investigation of philosophical matters, it will suffice to call it reasonable. As a general expression we may speak of the mind being variously impressed; and this is most convenient, for it would be cumbrous to speak of any mental impression as the mind existing in a separate state, which, however, the former phrase should of course be considered to imply. The mind cannot exist in two active states, or in a state composed of a plurality of activities, simultaneously. Whenever a person looks upon St. Paul's or any other edifice, he experiences an impression, or his mind assumes a passive state, a condition undetermined by any effort of his own; and immediately succeeding this, as he perceives that the impression is caused by the cathedral, which is the same thing as saying, as he perceives it to exist, which is the commencement of remembrance, his mind continues this active state: and as long as any one looked upon St. Paul's, an impression and perception, a passive and active state of the mind, would, I apprehend, be experienced at the same time. There is nothing in this doctrine which renders judgment, or any other mental operation, impossible. If I see half-a-dozen cubes, and am desirous of finding which is the largest, I have only, supposing the dimensional differences to be considerable, to look from one to the other in order to obtain general impressions of them all; when, if they are not far apart, I shall at once perceive which is the superior as regards size, or, if they are distributed over too large a surface for this, I shall be enabled to appeal successfully to the faculty of memory. The conclusion is formed by a number of comparisons, which are passed either upon the several general impressions existing simultaneously in the mind, or between the largest impression the mind has been cognizant of, and every other proceeding from the remaining cubes found in the journey of the eye, and which appear to be involuntary as far as vision reaches, that is, as far as difference of size can be perceived at the distances. The term perceived is here and before used in the sense we affix to the word distinguished, and it should be observed that this is not an error, inasmuch as the impression of a cube would not say anything as to its size unless we perceived it, or, which is the same thing, knew that this proceeded from a cube, and from experience were aware of its distance. We cannot have simultaneously general impressions of a large and small cube, or any other bodies, similar or different, either in size or form, neither can we remember bodies prior to perceiving others without comparison. Now, if this species of comparison is something involuntary, it cannot be set down as an active state of the mind; and even could it be made out to depend upon the human will and effort, this would not show that the mind sometimes exists in two active states simultaneously, and this because it could not be proved to be made while the percipient effort was put forth. Comparison is the knowledge of similarity or difference, and is often restricted to size, form, and colour. We have to deal only with perception and memory. When the mind simultaneously perceives the impressions of a number of cubes or other objects, we shall immediately know which is the largest and smallest, and become aware of all their other visually apparent distinctions. And so when we remember the largest impression the mind has received, and the eye in its movement is affected by new impressions, and thus is the cause of a series of mental conditions, a number of comparisons and conclusions, which are distinguished by the former being between only two objects, are the necessary mental results. Those who suppose that ideas are anything less than distinct states of the mind, will probably think it a strange doctrine which teaches that some comparisons and conclusions are involuntary, but I am convinced that experience establishes it. No man can look at a number of cubes or any other objects without knowing, if they are really different in size, which is the largest and smallest, and also, if the variations in these respects are sufficient, how they differ in form and colour. I see no reason for supposing these effects to be instinctive, but would submit that they are necessary and, of course, conscious conditions of the mind; for, of all the phenomena of the mind which direct a man, he must be conscious. Experience proves that perception is also involuntary. I cannot look at a church, a train, the desk before me, or the pen in my hand, without perceiving what realities the impression of these things denote, and this because remembrance, which is sometimes actively and sometimes passively produced, and in the latter case is known as association, and as regards perception is always passive, at once causes the mind to exist in the conditions of knowing what objects these impressions proceed from, which conditions subsist as long as the mental impressions, which may be called impressional conditions, accruing from the images on the retinas, are not destroyed. If a man is conscious of all the directing phenomena of his mind, it may be inquired, how is it that any difference of belief exists on any of these points? And this may be answered by directing the attention to certain mental phenomena, which appear to show that operations are performed, immediately forgotten, and afterwards denied. When a person has a number of objects before him, he, while gazing upon them, knows, if this is visually possible, which is the largest and which the smallest; or, to change this into philosophical language, his mind assumes a perceptional and an intellectual condition at the same time; and when one remembers the largest of a certain set of objects, and turns round to look at others, his remembrance—which is here something acquired, and thus a passive condition—enables him, if this is visually possible, to say whether the object of it is inferior in size to any of them. Between the object of his remembrance and every new impression which meets his eye, there is involuntarily on his part a comparison, or, philosophically, his mind exists in an intellectual, and thus in a plural state; and when he has looked at them all, it will, if no larger object has been found, return to its single state of the remembrance of the object, but if a larger has been seen, it will assume the condition of the simultaneous impression and perception of this. It has been said that perception is remembrance passively produced; and while no doubt may be had as to the correctness of this definition with respect to familiar objects, it may be thought not to apply where the objects are not understood. If a man sees something, the use of which he does not understand, but which he knows to be a piece of machinery, it cannot be said that, because he is ignorant of its use, he does not know that it is a piece of machinery. Neither, when a person sees a medal, upon which is inscribed an unknown bust and a sentence of an unknown tongue, and remembers to have seen such objects passed about from one to the other in foreign localities, can it be said that he does not know it to be some coin because he has not discovered its value. If we have the impressions of these things, or if our minds exist in these impressional conditions, we shall at the same time perceive them, and this although we do not know either the use of the one or the value of the other.

It has already been, I think I may say, shown by an eminent philosopher, that judgment of every kind is not a single active operation of the mind, but only a condition of it, resulting from previous active exertions. This explains how it is that some comparisons and conclusions are involuntary; certain objects produce corresponding impressional conditions, and simultaneously with this the mind assumes, apart from the will and effort of the possessor, an intellectual condition, a plural state of which he cannot but be conscious. The only active exertion here necessary is the corporeal one of turning to the objects; but as regards judgment at large, or general reasoning, several comparisons and conclusions are necessary before the final conclusion can be arrived at. And it is found that this conclusion is never a single active operation, but always the slipping, so to speak, of the mind into a new condition. Those who will inspect the operations of their minds when engaged in any species of reasoning, will find that every comparison and conclusion, to one of which particulars the several parts of every argument are easily deducible, is thus brought about; and therefore I shall not here offer any further remarks upon the question.

If it is allowed that the various mental ideas, by which I understand all knowledge derived from perception, in which may be included sensation and impression, and reflection, which term adequately implies every species of reasoning, or the totality of human knowledge, are nothing less than the mind successively existing in certain states, it may still be thought that no ideas, or very few ideas, are the mind in its entirety existing in that state which man is conscious of as the idea. And it must be confessed that this opinion is not unreasonable, especially when held by those who have been accustomed to materialize the mind by parcelling it out into various faculties. It is easy to see that the term mind is popularly used very vaguely. In common conversation people speak of their minds sometimes as meaning that which is distinct from their bodies, and sometimes with reference to their feelings only. It is true that the brain is what may be called the mechanical part of the mind, for here are all the several mental and moral organs which are set into activity by the living element itself; but, inasmuch as these organs are not self-exertive, and thus should not be called faculties except when operated upon by the living element, they cannot be set down as forming a part of the mind proper. I see now a number of books before me, and consequently believe that my brain is in some way affected; and knowing, moreover, that my brain is not myself, I further conclude that my mind is in connexion with the affected part or whole, and that by means of this I become aware of the impressions and perceptions. There is an awe in contemplating the meaning of the term Self which has not escaped the attention of man, and when fairly examined, few are content to believe that what each individual terms Myself is contained in the physical and palpable limits and materials of the human brain. Some who would admit that the impression of a house and all visional knowledge is mental, would begin to boggle if they were told that the sensation produced by contact with any hot or cold substance, or any other, is of the same character. But no real objection can be made to this assertion. The impression of a house or any other object is apprehended by the mind, and then perceived; and in like manner any sensation, as that of taste, is first apprehended and then perceived. It may be asked whether man cannot taste, feel pain, or experience any other sensation without the mind being affected, and to this a negative reply must be given. It is at once evident that a man devoid of every intellectual faculty could suffer, but that he could know he suffered, and much less, that he could comprehend his species of suffering, cannot, if we exclude the presence of instinct, be made out. When an animal sees its food, its memory informs it that the impression is that of its food, or, in other words, it perceives it, and this necessarily, as in the case of man. If there were a man devoid of every intellectual faculty who could not compare, and he was to suffer from a wound in the foot, for example, I cannot imagine that his want of intellect would in any degree abate the extent of his suffering; but, at the same time, I do not see how he could know that he suffered unless instinct were afforded him as a succedaneum for the former faculty, neither do I understand how he could tell whence it proceeded, and what was its nature. It requires no knowledge for a man to suffer; but to know that he suffers, and to ascertain any particulars respecting his suffering, are verbal implications of it. Now, all knowledge is either mental or instinctive, and consequently, apart from both mind and instinct, a person could only suffer or receive pleasurable impressions or sensations. The impression of a landscape upon the retina of a person devoid of mind would probably be gratifying, just as the sensation of a heated piece of metal upon the palm of the hand would be painful; but further than this, these things would not affect him. It cannot, I apprehend, be said with certainty that, as regards animals, instinct is anything more than desire. The bee builds her cells in a certain form, and the beaver constructs his habitation in a certain manner, and to explain these things it is not absolutely necessary to suppose that these creatures know that what they do is best; for, with the desire merely so to act, their purposes would be fulfilled. But as regards those operations of man which become instinctive, it appears that knowledge always accompanies desire. When a person proceeds to play a well-known tune upon any instrument, he not only desires to strike certain keys, or to press or vibrate certain cords, but he knows that these actions will produce the required harmony. And so an artificer or mechanic goes through his accustomed work, knowing that the various processes he is desirous of accomplishing will lead to the final result in view, which knowledge arises from instinct, as does also the desire. Of course, both the musician and the workman are desirous, as a part of their natural feelings, of accomplishing a certain result; but the various intervening desires conducive to these ends are either properly instinctive, or such as arise from instinctive knowledge. It is easy to be convinced that the mental part of practice, which is habit or association, at last gives way to instinct; for, if we pause during the performance of any of our habitual operations, we are unable to discover that they have been guided so far by the association of ideas, or any mental faculty. If instinct is allowed to be the mind in certain passive states, which direct the possessor and yet do not rise into consciousness, we must inquire what rising into consciousness means. Of instinct, as effects, we are conscious, and consequently man can be called unconscious of instinct only as regards its cause. But this unconsciousness does not interfere with the fact that instinct is known as effects, and consequently the definition is incorrect.

I have just taken mine eyes from off a range of hills, on which I saw trees scattered and growing in darksome masses, and cultivated land; and make no doubt that I had but a general mental impression of the scenery, and at the time had no knowledge of my mind but that it existed in that impressional condition, and immediately afterwards perceived what it indicated, or became aware of the existence of an external landscape. It does not follow that because when mentally apprehending and perceiving physical impressions and sensations, we are not aware of the existence of the mind in any other way, that what we are conscious of is the mind in its entirety, neither that any active or pensive mental condition, which is all we know of the mind at the time, is itself in its completeness: and thus the question must be considered in an indirect manner. It is evident that man is always conscious of his mind as far as he can be conscious of it, because it is not in his power to be ignorant of this condition at the time, although he may soon erase it from his memory; and unless the mind is thought to be parcelled out into a number of faculties, which should properly be said to compose the brain, the idea of the unconscious part, if such is supposed, cannot be very erroneous. I do not find positive evidence against supposing that the mind proper is anything more than the soul, or that element which is termed life, which may set into activity the various intellectual and moral organs of the brain, and the simplicity or compoundedness of which cannot, I think, be determined. The mind may be a separate element, and this either simple or compound: if the former, then when I perceive a tree, or am conducting some intricate piece of reasoning, or my mind exists in any other state, I shall consider it probable that the whole of my mind proper exists in the condition of which I am conscious; but if the mind is compound, that is as before if I so consider it, I shall suppose that some of its parts are not in a state of which I can and must be conscious, and hence do not affect my knowledge, but that these will be experienced when connected with the brain, just as invisible writing appears when brought into contact with water or other substances. The question principally hangs upon determining whether or not the mind is anything apart from the element termed life, and whether in either case it is simple or compound: if the soul or life is set down as simple, and the mind proper, it will not, perhaps, be contradictory to affirm that we are always conscious of the whole of it, because as the life it may operate unconsciously to us; and if as compound the remarks made upon supposing the mind to be a separate and compound substance are applicable, and should be coupled with the former observation.

Many of the ideas arising in the mind may be accounted for upon the principle of association, but I do not hesitate to affirm, as the result of having carefully inspected those of my own experience, that nothing can be more erroneous than to insist that all can thus be explained: and in support of the assertion I appeal to the necessary experience of every individual. It may be said that in those cases where the principle of association cannot be found to apply, the various intervening links have been forgotten, and hence that the want of remembrance does not prove its absence, or that some unconscious mental changes have occurred, but I take both of these explanations to be abortive : every person has had many ideas come, or rather bolt, into his mind, if I may be allowed a somewhat vulgar though very forcible expression, which he has been unable to account for, and I hold it incredible that in the majority of these cases every intervening link of circumstance would have been forgotten, supposing that a connecting band of events had once existed. As regards latent or unconscious mental changes, I do not understand how the mind can contribute either to human knowledge or desire, without every step of the process being perceived, and am convinced therefore that those subjective ideas which cannot be explained upon the principle of association, are the mind existing in certain states, and that they absolutely demonstrate the truth of this doctrine, at any rate as regards this particular. And if it is applicable to certain mental ideas, there is much reason for supposing that it should be applied to every such condition. The notion that ideas of every kind are things in the mind is one which, though derived from physical phenomena, cannot be pronounced absurd : to say that the idea of a tree is something in the mind is similar to affirming that a pebble is something in the hand ; and while as a doctrine reasonable, although I hold it to be unable to meet the above phenomenon, it must, I think, yield as such in probability to the other, to which no objection can be raised, because it is necessary to suppose that the mind turns very rapidly into different states, seeing that being acquainted only with matter, which is lumpish at the best, we do not know what cannot be affirmed of mind or spirit. A particular attention should be given to those ideas which cannot be accounted for by the principle of association, and this inasmuch as it may be concluded that they are the mind existing in various states, which variations may be likened to certain india-rubber resemblances of human and other faces, which, by being variously squeezed, are changed as to contour, the amount of matter in every case being the same. I may be engaged at my devotions, and perhaps suddenly there will start up before me various what may be called pictorial ideas (imaginings) ; as, for example, a part of a town which I had visited, or the form of a friend or acquaintance in a situation where I had seen him. Now, if upon connecting these or other ideas with the previous objects of my thoughts, I could not with the closest attention find even a single link by which the presence of the associative principle could be concluded, it would be philosophical to conclude that it had not existed, and moreover that the ideas were the mind existing in certain states. If this explanation is denied it is not possible to know what they are, seeing that they do not reach us by any sensuous inlet, not being either impressions from without, or things created by them by means of any of our physical organs. The occurrence of the ideas of dreams without the exertion of memory must also, where no link of association can be discovered, be set down as the mind existing in certain states.

The expression to have thoughts or words at the fingers' ends is a common one, and means that the thoughts or words in question would be known, and this immediately, to be the correct ones, upon their arising in the mind, or, which is the same thing, the individual becoming conscious of or possessing them. The term thoughts is popularly loosely used, and here stands for all possible ideas and every process of reflection, or everything of which the human understanding is susceptible, in consequence of which it should be thrown aside in philosophical writing, which I shall do. It is of great importance to observe that all our ideas are the appearances of real or imagined objects or phenomena, and this however they are brought about. The sentences "The calm sea," or "The grey hills," are composed of words which, if properly uttered, are preceded by the appearances of these objects, whether or not they are imagined, or in any way are reproduced in the mind with more or less accuracy, after having been actually inspected ; and similarly the words "I am hungry," or "I will take a journey," are occasioned by the idea of a hungry person consequent upon the sensation of hunger, and the general idea (appearance) of what may compose the taking of a journey, the general appearance being of course more or less imperfect, according to the mental and moral nature of the individual, and never complete. It does not require much attention to find that most of the words made use of by man, the principal parts of our ordinary discourse, are regulated merely by habit, it not being known whether or not they are the things which should have been spoken, or, which is the same thing, the sense of rightness not arising, or, when this is the case, their meaning not being considered. These two points are very important ones : it would be easy for any individual to catch himself using words and phrases merely habitually : and when he has found that he did not know them to be correctly employed, he may afterwards use them without previously having had corresponding ideas, or, which is the same thing, without having known and knowing their meaning. I wish the word "may" to be understood in the sense of possibility.

After the mind has been assiduously employed in any process of reflection or imagination, various ideas both apparent and reflective, that is various pictorial and reflective ideas, spontaneously occur or are suggested, the latter of which are of course reflective only in the passive meaning of the term, that is as passive operations of the mind: now, if any analogy may be supposed between the nature of mind and that of matter, a question upon which I shall not offer an opinion, it may be supposed that this involuntary activity is caused somewhat in the same way as momentum is produced in matter, the after working of the mind resembling the motion of an object when the force which caused it to move has been taken away. This hypothesis explains why these ideas cannot be got rid of without much difficulty, and is, I take it, as well founded as are any of the kind: however, I shall not further make use of it, as dwelling upon hypotheses is but rickety work, especially those which relate to the mind and its analogy or resemblance to matter.

It is evident that the knowledge man acquires by experience, cannot be increased without the application of the various rules of reason. And as man has obtained much knowledge, which the mere perception of the various objects of nature would never have conveyed, it may be concluded that he has both discovered and applied the real principles of reason. Mere ocular perception is sufficient to inform man that a tree undergoes far more rapid and somewhat more uniform change than a mountain, but by this alone it could never be known that these bodies belong to different classes of objects, the one to the vegetable and the other to the mineral kingdom. Other senses must be exerted before this knowledge could have been obtained. This illustration shows that the various senses of man must be exercised, that our several perceptive faculties must be employed, before we get all that knowledge which it is possible to obtain apart from reason; but no principle of reason is connected with it. When a stone is dropped from the hand it is found to fall with increasing velocity, but the mere knowledge of this fact would never teach man the laws of gravity, or even the laws of motion. It requires at least two experiments to infer that bodies always fall with increasing velocity, and also two to know that the attraction which one body exercises upon another varies inversely as their distance apart. Thus mental inferences must be added to mere perceptions before much knowledge of a physical or any other kind can be obtained. It will be understood that two observations in both of the above cases are absolutely necessary, and that a greater number are useful for the confirmation of the inferences thereby obtained. The question of gravity has been supposed as concerns this world only; and it is more evident that mental inferences are necessary, when it is extended to the universe generally.

It is allowed that every species of reasoning should be as simple as possible; and to this end men should strive after logical accuracy without the use of logical forms. The want of logical accuracy is most experienced in moral questions, and discussions of a political or social nature; and next to these, the various systems of religion present the most objections in this respect. In all these considerations logical forms are very seldom made use of, being almost restricted to mathematics, and the higher branches of the other sciences. The syllogism is a logical instrument, concerning which it appears to me that much confusion both has existed, and is now to be found. It has been and is now supposed that we learn nothing by it, and it is looked upon as merely the grand lever of scholastic disputation, and from both points as a something which cannot now be of any use, and must only be called a curiosity. I propose to make a few observations upon this view of the question.

Suppose a woodman, without having experienced the effects of fire, to stumble upon the remnants of one lately kindled: now, inasmuch as we have supposed him ignorant of these effects, he could never of himself account for what he witnesses. Suppose him also to take up and examine a piece of charcoal, and after this to meet with the remains of another fire: finding there substances similar to the substance he had before examined, he would infer, that whatever produced the latter was also the cause of the former. Although very improbable that any in such circumstances would so express themselves, I cannot but think, that his inference would mentally be grounded upon the following proposition: Every similar effect must have a similar cause: the substance I have examined is similar to those substances now before me, and hence must have been produced by a similar cause. It is evident that this process would, after the first time, become habitual or instinctive; and from this consideration it cannot be objected, that mankind thus think when they commence to reason. Man is rightly considered a creature of habit; and this condition it is which makes him forget those principles of reason which made him acquainted with truths, which ever since have habitually occurred to his mind. Habit may at first sight lead many to imagine, that they see certain links in an intuitive or inoperative manner; but this supposition is mystical, unsupported by facts, and contrary to all philosophical analogy. It may be, and I think has been, supposed that we perform mental operations without being conscious of this; but, except as to taking for granted certain things, which are operations so familiar as generally to escape us, but which, it is only rational to suppose, must in every case be made by habit or association, I apprehend that we are conscious of every direct or proximate as distinguished from habitual or associative operation. It may be thought that the human mind can never operate, even habitually, without this being perceived. If a man watches the formation of a medal, and afterwards sees another of a similar nature, he will at once conclude that the latter was fashioned after the same manner as the former; and here it may be said that he does not suspend his judgment to consider whether similar effects must be owing to similar causes. I think that a nice attention to the various states of the mind during this or any other judgment, would show that this proposition is always tacitly and habitually assented to: without this I do not see upon what ground man could proceed with any argument; and I think that the individual in question, however inferior or imperfect his parts, would be conscious of a flaw somewhere. The syllogism in question appears to me something necessary to every species of argument, unless the inference can be obtained in some other way, which I can find no reason for supposing; and consequently it appears to me, and this in common with its application universally, an instrument of knowledge or discovery.

It is not necessary that various syllogisms should be formally pronounced, in order to their being the substrata of all human reasoning; and because this is the case, it should not be supposed that the propositions are not mentally assented to. As regards the various axioms and definitions connected with mathematical science, which are assented to as soon as proposed by all who understand the meanings of the terms in which they are expressed, it may be granted that no syllogism is necessary. Man has here to deal merely with abstractions, and consequently it is not necessary to have recourse to those forms which are necessary when judging of matters depending upon experience. The assertions, a whole is greater than its part, or, two straight lines cannot enclose a space, are evident as soon as the meanings of the terms in which they are cast are known; and the same thing may be said of all propositions which exclude experience. But in all other sorts of reasoning, I suppose that the syllogism is, and this in every particular instance, mentally employed in an habitual manner. The ideas and feelings of man are regulated by association, and here there is some analogy for supposing that our mental operations are thus induced, or occur by habit. Suppose the following proposition in moral philosophy to gain any man's assent—Utility is the object of all human actions, therefore it should be the principle influencing every man's conduct. Let it be noticed that things are here assumed, or rather, tacitly assented to. It is assumed that man has, at any rate, one other principle of action, and that this is a wrong one, and that all human actions have an object, and are performed from some principle. And from these points flow necessarily, I think, the following syllogisms :—A plurality of principles characterize human actions; but one principle can be correct, therefore the remainder are harmful. And again—Various objects dictate human actions; one alone can be proper, therefore the remainder are improper, or unproductive of use. It should not be thought that there is no distinction between a man's object, and the principle which moves him in a particular action. The object of a man may be the destruction of the birds or noxious animals which infest his grounds, whereas the principle dictating this may be merely the endeavour after a little amusement. I cannot but think that the points and syllogisms alluded to would habitually be presented to the mind, and be assented to with instinctive readiness, although the process is strictly mental, whereas the above proposition is presented to the mind of any individual. And, for the reason given, I answer that similar points and syllogisms would, in like manner, as regards every mind, precede the assent to all other propositions grounded upon experience, whatever their nature.

The physical sciences are generally said to grow by experience, so that to talk of the syllogism as an instrument of scientific discovery, may be thought carrying the human intellect many centuries backwards. I am, however, apprehensive that the use of the syllogism underlies all our knowledge, even of this sort. Suppose a man, in an unknown country, to discover some dust of gold in a particular stratum of a certain mountain, or the sand of a certain river. He may be no geologist and know nothing of the science, yet from analogy he might suppose that the same substance was to be found in the same stratum, or similar strata, elsewhere; he would at any rate suppose this more probable than the fact that it could be found elsewhere in a different stratum or various other strata: and his opinion on these points would be strengthened if he continued to find more of the same substance in the same stratum, particularly if not in the same locality. The same reasoning applies to the sand of the river, where analogy likewise introduces itself. The least consideration will make it clear that no syllogism operates in either case, and that it is incompatible with mere analogy. Analogy is merely a certain disposition of the mind, when certain particulars are set before it, and further than this will never perhaps be accounted for. All we here have is this, a certain substance is found under certain conditions, and it is supposed that more of it will be found under similar conditions; or, at any rate, that this is more probable than that it exists under other circumstances. I do not see that any proposition can be got out of this; it does not appear capable of being thrown into the syllogistic form, nevertheless, I take it to depend upon the syllogism, showing that similar real effects must be owing to similar real causes. A certain stratum has been found to contain gold, and it is supposed that other strata of the kind contain it also, because, inasmuch as all strata of the same kind must be supposed to have been formed alike, it is probable that the condition of all is similar. It should be observed, that we have here supposed something more than analogy proper. When a man meets with substances of the same nature, the question of analogy, as to their physical condition, cannot enter his mind; he perceives that they are similar; but why, because one is found to contain some substance, he should consider it probable, and perhaps certain, that the rest contain it, it is not easy to discover. I am now supposing a person wanting experience as to the condition of the terrestrial rocks and mountains; and even were not this the case, it is almost, if not quite as difficult to understand, how the mental disposition known as analogy is produced. It is useless to mention any human operations or contrivances as illustrative of it, because we know, or may readily guess, of what these consist. If I see a church in the course of erection, I consider it in the highest degree probable, from past experience, that it is intended to be finished; and, similarly, if I see a certain medal, and am told that it has a centre of bronze, which is covered over to escape detection, and am shown another of a similar mould, and, according to appearance, of similar nature, I think it in the highest degree probable, that it is really similar to the former. As regards human affairs, man soon becomes acquainted with the general practices and dispositions of man, and hence is enabled with tolerable certainty to judge of his future actions; but no experience of this kind can, from the nature of the thing, be obtained from the physical world. It may be observed, how the various treasures of nature are distributed, but nature being a collection of unimpressible materials, we cannot, of course, obtain experience from her as we can from man. Man operates, while nature has already received every important arrangement it ever will have been the subject of, that is, of course, as regards the present mundane state of things.

It appears to me that we must advance a little farther before another syllogism can be supposed to direct the judgment.

Imagine the man to have a specimen of gold dust about him, and, upon meeting with the same substance in the rock, to compare it with this; he would then probably, from the mere contemplation of their apparent qualities, pronounce these substances similar. Upon finding more of some substance elsewhere, also resembling the gold dust in his possession, he would conclude it similar to the substance first discovered; and this I apprehend by means of the following proposition: the two assortments of substance found resemble that in my possession: things which are equal to the same things are equal to one another; therefore, these substances are at any rate similar as to their apparent qualities. He might not conclude that either of the substances was similar to that substance he possessed; and even if he supposed that the one was, this idea would not be rational, inasmuch as they are supposed similar in their apparent qualities, which comprise everything man can judge by, on which account he would have no reason for supposing the one completely similar to another substance, the internal qualities of which he might know, in opposition to the other. It may be said that the memory must enable a man to know whether substances thus discovered were similar, and hence that the comparison and consequent syllogism is not absolutely necessary: this must be granted; nevertheless, I think that when the comparison is made, the proposition connected with it in these observations must be gone through, to render it productive of any new truth. If this supposition is correct, it is evident that this mental proposition occurs so habitually as to escape detection by any but the closest attention: and the same thing must be said of every other syllogistic formula which embodies a proposition, the truth of which at first sight appears independent of reason. I apprehend a near inspection will eventually convince the philosophical community, that all our most evident truths are founded upon various propositions, all of which commonly escape attention. As an abstract truth, nothing is more evident than that a whole is greater than its part, yet when practically considered, I think that this proposition must assume the shape of a syllogism, that is, of course, when looked at apart from experience. Were I to tell a man born blind, who had of necessity never seen, and might never have felt an orange, that the whole of one is greater than a part of the same, he would at once assent to this as perfectly in accordance with reason, and this, I think, by the aid of the following proposition: abstractedly, and therefore really or practically, a whole is greater than its part: an orange is a whole, and anything short of this a part; therefore an orange is greater than any portion of one. Supposing him to see or feel an orange, and understand the meaning of the term part, he would assent to the above statement merely upon the evidence of his senses, for it is obvious that no proposition need in this case be considered; but where this evidence is wanting I do not see how the syllogism is to be evaded, if a rational assent to any true statement is undertaken; and it appears to me that the same thing applies to the denial of any assertion, the syllogism being here also used as an instrument of knowledge or judgment. The above remarks apply to the proposition, two straight lines cannot enclose a space; and other assertions, considered in their practical application. The abstract proposition, nothing can exist and be annihilated simultaneously, is evident as soon as the words in which it is expressed are understood; but when embodied in some objects of experience, I think that the syllogism is necessary to render it evident. Man exists: nothing can exist, &c.; therefore, man is not annihilated. As in the former example, we must here suppose direct experience to be excluded. When a man sees or takes hold of another, it certainly requires no logical formula to prove that he exists, and, consequently, is not annihilated; neither to infer that this condition is universally applicable, or the abstract proposition; but when a man is merely described, I suppose we do not intuitively pounce upon the fact that he is not annihilated. I cannot believe anything between this and the use of the syllogism; and inasmuch as there is much against the notion of anything being got after the former fashion, particularly in cases of this kind, I apprehend that a logical formula, unexperienced on account of its constant recurrence, is the instrument of our knowledge. It may be remarked, that a dissent to the proposition under consideration implies a contradiction in terms: this is true, but it is the very thing, the reason for our belief in which is here considered. Why do we believe that a denial of the proposition is a contradiction in terms? In other words, why do we consider it true? The assertion, whatever it is, is too rickety for my comprehension; it seems to me a notable specimen of logical tautology; for, look at it as much as I will, I cannot twist it into any sort of proposition.

If it is true that various syllogisms underlie all human reasoning dependent upon experience, which seems to me the case, it is important that this should be known, and more so, that these propositions should be logical in all their parts. I apprehend that this latter requirement is fulfilled; but when we come to the obvious conclusions of civil society, we continually find the quasi syllogism made the stronghold of doctrines and practices, alike foolish and mischievous. Examples of false syllogisms may be found in every logical treatise. As to those supposed, their simplicity and habitualness may be taken as conditions guaranteeing their truth.

It is very necessary to observe in connexion with this subject, that many of our actions are performed apart from any species of thought, being dependent merely upon the inclination. When a man hears some person call him he turns round or looks forward because he desires it, and this without considering whether or not he has the power to turn his neck or move his body; or when one is coming out of a place of worship and puts on his hat, he has not previously considered whether or not he can lift his arm. No person in either of these or any similar circumstances thinks whether similar appearances indicate similar realities, but every one is guided merely by his inclination. But whenever a man harbours any reasoning process, however simple, I take it that some syllogism forms the basis of it. Many persons may say that it is impossible that any person considers any syllogism as a part of any reasoning process and is not aware of it; and no remark can be more philosophical; but if any one should go on to remark, that because this process is not remembered afterwards it never has been performed, we have a right to inquire how this follows. If we inspect our thoughts I think we may be conscious that the syllogism is considered, and find that the remembrance of this consideration is immediately lost, and if so, it may be concluded, that those who think that no syllogisms underlie obvious human reasoning, only forget, by not noting at the time what they have often experienced. Some may suppose that the knowledge which appears to arise from various syllogisms is obtained by means of instinct, but this, for the reason given, I am unable to agree with. That instinct does play some part in human affairs I am persuaded, but it cannot be too forcibly said, that to resort to it while we are able to perceive any effort of reason, is both indolent and unphilosophical.

---

